# IL-1RA promotes oral squamous cell carcinoma malignancy through mitochondrial metabolism-mediated EGFR/JNK/SOX2 pathway

**DOI:** 10.1186/s12967-023-04343-9

**Published:** 2023-07-17

**Authors:** Shyng-Shiou F. Yuan, Yun-Ming Wang, Leong-Perng Chan, Amos C. Hung, Hieu D. H. Nguyen, Yuk-Kwan Chen, Stephen Chu‐Sung Hu, Steven Lo, Yen-Yun Wang

**Affiliations:** 1 Graduate Institute of Medicine, College of Medicine, Kaohsiung Medical University, Kaohsiung, 807 Taiwan; 2 Department of Medical Research, Kaohsiung Medical University Hospital, Kaohsiung, 807 Taiwan; 3 Department of Obstetrics and Gynecology, Kaohsiung Medical University Hospital, Kaohsiung, 807 Taiwan; 4 Translational Research Center, Kaohsiung Medical University Hospital, Kaohsiung, 807 Taiwan; 5 Drug Development and Value Creation Research Center, Kaohsiung Medical University, Kaohsiung, 807 Taiwan; 6 Department of Biological Science and Technology, Institute of Molecular Medicine and Bioengineering, Center for Intelligent Drug Systems and Smart Bio-devices (IDS2B), National Yang Ming Chiao Tung University, 75 Bo-Ai Street, Hsinchu, 300 Taiwan; 7 School of Dentistry, College of Dental Medicine, Kaohsiung Medical University, No.100, Shih-Chuan 1St Road, Sanmin Dist., Kaohsiung, 80708 Taiwan; 8 Cohort Research Center, Kaohsiung Medical University, Kaohsiung, 807 Taiwan; 9 Faculty of Medicine, College of Medicine, Kaohsiung Medical University, Kaohsiung, 807 Taiwan; 10 Department of Otorhinolaryngology-Head and Neck Surgery, Kaohsiung Municipal Ta-Tung Hospital and Kaohsiung Medical University Hospital, Kaohsiung, 807 Taiwan; 11 Division of Oral Pathology & Maxillofacial Radiology, Kaohsiung Medical University Hospital, Kaohsiung, 807 Taiwan; 12 Department of Dermatology, College of Medicine, Kaohsiung Medical University, Kaohsiung, 807 Taiwan; 13 Department of Dermatology, Kaohsiung Medical University Hospital, Kaohsiung, 807 Taiwan; 14 Canniesburn Regional Plastic Surgery and Burns Unit, Glasgow, G4 0SF UK; 15 College of Medical, Veterinary and Life Sciences, University of Glasgow, Glasgow, G12 8QQ UK

**Keywords:** IL-1RA, Mitochondrial metabolism, EGFR, JNK, SOX2, Cancer stemness, Tumor growth, Metastasis, OSCC

## Abstract

**Background:**

Interleukin-1 receptor antagonist (IL-1RA), a member of the IL-1 family, has diverse roles in cancer development. However, the role of IL-1RA in oral squamous cell carcinoma (OSCC), in particular the underlying mechanisms, remains to be elucidated.

**Methods:**

Tumor tissues from OSCC patients were assessed for protein expression by immunohistochemistry. Patient survival was evaluated by Kaplan–Meier curve analysis. Impact of differential IL-1RA expression on cultured OSCC cell lines was assessed in vitro by clonogenic survival, tumorsphere formation, soft agar colony formation, and transwell cell migration and invasion assays. Oxygen consumption rate was measured by Seahorse analyzer or multi-mode plate reader. PCR array was applied to screen human cancer stem cell-related genes, proteome array for phosphorylation status of kinases, and Western blot for protein expression in cultured cells. In vivo tumor growth was investigated by orthotopic xenograft in mice, and protein expression in xenograft tumors assessed by immunohistochemistry.

**Results:**

Clinical analysis revealed that elevated IL-1RA expression in OSCC tumor tissues was associated with increased tumor size and cancer stage, and reduced survival in the patient group receiving adjuvant radiotherapy compared to the patient group without adjuvant radiotherapy. In vitro data supported these observations, showing that overexpression of IL-1RA increased OSCC cell growth, migration/invasion abilities, and resistance to ionizing radiation, whereas knockdown of IL-1RA had largely the opposite effects. Additionally, we identified that EGFR/JNK activation and SOX2 expression were modulated by differential IL-1RA expression downstream of mitochondrial metabolism, with application of mitochondrial complex inhibitors suppressing these pathways. Furthermore, in vivo data revealed that treatment with cisplatin or metformin—a mitochondrial complex inhibitor and conventional therapy for type 2 diabetes—reduced IL-1RA-associated xenograft tumor growth as well as EGFR/JNK activation and SOX2 expression. This inhibitory effect was further augmented by combination treatment with cisplatin and metformin.

**Conclusions:**

The current study suggests that IL-1RA promoted OSCC malignancy through mitochondrial metabolism-mediated EGFR/JNK activation and SOX2 expression. Inhibition of this mitochondrial metabolic pathway may present a potential therapeutic strategy in OSCC.

**Supplementary Information:**

The online version contains supplementary material available at 10.1186/s12967-023-04343-9.

## Background

Oral cancer is one of the most prevalent cancers and causes of cancer-related deaths worldwide, and oral squamous cell carcinoma (OSCC) is the most frequently diagnosed form of oral cancer [1–3]. Various risk factors promote oral cancer development, including tobacco use, alcohol consumption, and betel quid [4]. However, the management of oral cancer remains a challenge owing to its tendency for frequent relapse, metastasis, and treatment resistance [4–6]. An important potential mechanism underlying some of these oncological challenges is the property of cancer stemness, which is associated with the enhanced self-proliferation and metastatic ability of cancer cells. Recent research has suggested that altered mitochondrial metabolism underpins some of these malignant cell characteristics [7–10].

The interleukin-1 (IL-1) family includes both pro- or anti-inflammatory proteins, with pro-inflammatory IL-1β being the best characterized one [11]. On the other hand, interleukin-1 receptor antagonist (IL-1Ra) is a natural anti-inflammatory antagonist of the interleukin-1 family of pro-inflammatory cytokines [12]. IL-1RA, the gene product of *IL1RN* which shares approximately 30% protein sequence homology with interleukin-1 (IL-1), competes with IL-1 for binding to the IL-1 receptor [13, 14]. As a member of the IL-1 family with multifaceted functions, IL-1RA plays diverse roles in a number of pathological conditions, including cancer. For example, lower levels of IL-1RA were found in several cancer types, such as leukemia, colorectal cancer, and prostate cancer [15–17], and it was negatively associated with the development of premalignant oral dysplasia [18]. However, other reports indicate higher levels of IL-1RA in cervical and gastric cancers [19, 20], suggesting that IL-1RA may function in a cancer type-specific manner. However, its clinical role and biological function in oral cancer development remain unclear. Accumulating evidence has linked the development of oral cancer with IL-1-mediated chronic inflammation [21]. It is intriguing to investigate whether IL-1RA, an anti-inflammatory antagonist of IL-1, also plays a role in oral carcinogenesis.

In this study, we evaluated the potential of IL-1RA as a clinical marker for OSCC progression and patient outcomes. In addition, the cellular mechanisms of IL-1RA in OSCC malignancy were explored both in vitro and in vivo, providing insights into the development of novel therapeutic strategies for OSCC that target vulnerabilities in the IL-1RA-associated mitochondrial metabolic pathway.

## Methods

### Patient samples

Oral tumor tissues were obtained from patients diagnosed with OSCC at the Department of Oral and Maxillofacial Surgery, Kaohsiung Medical University Hospital, Kaohsiung, Taiwan, and confirmed using clinical and histological data from the Cancer Registry. Overall survival was defined as the interval between the date of diagnosis and date of death. This study was approved by the Institutional Review Board of Kaohsiung Medical University Hospital (approval no. KMUH-IRB-20130300 and KMUHIRB-F(I)-20220016). Patient informed consent was waived by the Institutional Review Board due to the retrospective nature of the study.

### Cell culture

Human oral squamous cell carcinoma cell lines HSC-3, Ca9-22, and OECM-1 were obtained from the Bioresource Collection and Research Center, Hsinchu, Taiwan (https://www.bcrc.firdi.org.tw). HSC-3 and Ca9-22 cells were cultured in DMEM/F12 medium (Thermo Fisher Scientific, Waltham, MA, USA), and OECM-1 cells were cultured in RPMI 1640 medium (Thermo Fisher Scientific, Waltham, MA, USA). All cell culture media were supplemented with 10% fetal bovine serum (FBS; Biological Industries, Beit Haemek, Israel) and 1% penicillin–streptomycin-amphotericin B (Thermo Fisher Scientific, Waltham, MA, USA). Cells were maintained at 37 °C in a humidified incubator with 5% CO_2_ incubator.

### Gene knockdown and overexpression

Knockdown of IL-1RA was conducted by lentiviral infection using a pLKO.1-puro vector carrying shRNA sequences (shIL-1RA#1, 5’-GCCTTCAGAATCTGGGATGTT-3’; shIL-1RA#2, 5’-CGAGAACAGAAAGCAGGACAA-3’; shIL-1RA#3, 5’-GCAAGGACCAAATGTCAATTT-3’; shIL-1RA#4, 5’-CGTCATGGTCACCAAATTCTA-3’) that target consensus regions of human *IL1RN* (Accession: NM_000577), which were obtained from the National RNAi Core Facility, Academia Sinica, Taipei, Taiwan. Another pLKO.1-puro vector carrying shRNA sequences targeting firefly luciferase (shLuc, 5’-GCGGTTGCCAAGAGGTTCCAT-3’) was used as a control (National RNAi Core Facility, Academia Sinica, Taipei, Taiwan). For gene overexpression of IL-1RA, prepackaged lentiviral particles carrying pReceiver-Lv105 vector expressing full-length human *IL1RN* (IL-1RA-OE; Accession: NM_173841.2), or empty pReceiver-Lv105 vector (EV) as a control, were purchased from GeneCopoeia (Rockville, MD, USA). The infection was performed by adding lentiviral particles to the corresponding cells in the cell culture medium containing 8 µg/mL polybrene (Sigma-Aldrich, St. Louis, MO, USA). After infection for 48 h, 2 µg/mL puromycin was added for selection, and the surviving cells were maintained in 2 µg/mL puromycin until further experiments.

### Clonogenic survival assay

OSCC cells were seeded in 6-well plates (1 × 10^3^ cells/well; Corning, Tewksbury, MA, USA) and cultured for 14 days prior to staining with 0.5% crystal violet (Sigma-Aldrich, St. Louis, MO, USA) for 15 min at room temperature. For ionizing radiation treatment, cells were irradiated at a dose of 5 Gy using a 6-MV linear accelerator (Elekta, Stockholm, Sweden) at the Department of Radiation Oncology, Kaohsiung Medical University Hospital, Kaohsiung, Taiwan. Images were captured using a light microscope (Nikon, Tokyo, Japan) and analyzed using ImageJ software (https://imagej.nih.gov/ij).

### Tumorsphere formation assay

OSCC cells were seeded in ultra-low attachment 96-well plates (5 × 10^2^ cells/well; Corning, Tewksbury, MA, USA) in serum-free cell culture medium supplemented with 20 ng/ml epidermal growth factor (EGF; PeproTech, Rehovot, Israel), 20 ng/ml fibroblast growth factor-basic (FGF-basic; PeproTech), 10 µg/mL insulin (Sigma-Aldrich, St. Louis, MO, USA), and 1X B27 (Thermo Fisher Scientific, Waltham, MA, USA). After 14 days of cell culture, images of tumorspheres larger than 50 μm in diameter were captured using a light microscope (Nikon, Tokyo, Japan) and analyzed with ImageJ (https://imagej.nih.gov/ij).

### Soft agar colony formation assay

OSCC cells were suspended in cell culture medium mixed with 0.4% low-melting agarose (Sigma-Aldrich, St. Louis, MO, USA) and then seeded in 6-cm dishes (1 × 10^4^ cells/dish; Corning, Tewksbury, MA, USA) pre-coated with a layer of 0.5% low-melting agarose (Sigma-Aldrich, St. Louis, MO, USA). After 14 days of cell culture, colonies larger than 100 μm in diameter were stained with 0.5% crystal violet for 15 min at room temperature, and images were captured using a stereo microscope (Olympus, Tokyo, Japan) and analyzed with ImageJ (https://imagej.nih.gov/ij).

### XTT cell viability assay

OSCC cells were seeded in 96-well plates (1 × 10^4^ cells/well; Corning, Tewksbury, MA, USA) for 24 h, prior to the addition of XTT reagent from Cell Proliferation Kit II (Sigma-Aldrich, St. Louis, MO, USA) for 2 h at 37 °C, followed by measurement of the optical density (OD) at 470 nm by subtracting the background at 660 nm.

### Transwell cell migration and invasion assays

OSCC cells were re-suspended in serum-free cell culture medium and plated onto inserts (2 × 10^4^ cells/insert; 8 μm pores) in 24-well transwell plates (Corning, Tewksbury, MA, USA). The inserts were pre-coated with or without Matrigel (Corning, Tewksbury, MA, USA) for cell invasion and migration assays, respectively, and normal cell culture medium was added to the bottom wells. After 24 h, cells that remained on the upper side of the inserts were removed using cotton swabs, and cells that appeared on the lower side of the inserts were fixed with 4% formaldehyde (Sigma-Aldrich, St. Louis, MO, USA) for 15 min and stained with 0.05% crystal violet (Sigma-Aldrich, St. Louis, MO, USA) for 30 min at room temperature. Images were captured using a light microscope (Nikon, Tokyo, Japan) and analyzed using ImageJ software (https://imagej.nih.gov/ij/).

### Measurement of oxygen consumption rate (OCR) and extracellular acidification rate (ECAR)

The basal OCR and ECAR of OSCC cells were measured using the Extracellular Oxygen Consumption Assay and Glycolysis Assay kits (Abcam, Waltham, MA, USA), respectively, according to the manufacturer’s instructions. The fluorescent signal was measured using a CLARIOstar Plus multi-mode plate reader (BMG Labtech, Ortenberg, Germany) at 1.5 min intervals with excitation/emission wavelengths of 360/650 nm for OCR and 380/615 nm for ECAR. To measure multiple statuses of OCR in mitochondria, an Agilent Seahorse XFe24 Analyzer (Agilent Technologies, Santa Clara, CA, USA) was used in accordance with the procedures from the manufacturer and our previous report [22]. The mitochondrial modulators were obtained from Seahorse XF Cell Mito Stress Test Kit (Agilent Technologies, Santa Clara, CA, USA) and injected sequentially for specific measurements as follows: first, 1 μM oligomycin was injected to measure ATP production; second, 0.5 μM carbonyl cyanide 4-(trifluoromethoxy)phenylhydrazone (FCCP) was injected to measure maximal respiration; finally, 0.5 μM rotenone and 0.5 μM antimycin A were injected to measure spare respiratory capacity [23].

### Measurement of reactive oxygen species (ROS) formation

OSCC cells were seeded in 6-well plates (3 × 10^5^ cells/well) overnight, followed by labeling with 10 μM 2’,7’-dichlorofluorescin diacetate (DCFDA; Sigma-Aldrich, St. Louis, MO, USA) for 20 min at room temperature. The cells were then analyzed using a Cytomics FC 500 Flow Cytometer (Beckman Coulter, Indianapolis, IN, USA) in accordance with the manufacturer’s instructions and our previous report [22].

### Polymerase chain reaction (PCR) array

Total RNA extracted from OSCC cells was reverse transcribed and analyzed by real-time PCR using the Human Stem Cell Signaling RT^2^ Profiler PCR Array (Qiagen, Redwood City, CA, USA) according to the manufacturer’s instructions and our previous report [24].

### Proteome array

Total protein extracted from OSCC cells was analyzed using the Human Phospho Kinase Array (R&D Systems, Minneapolis, MN, USA), according to the manufacturer’s instructions and our previous reports [25, 26].

### Western blot

Total protein extracted from OSCC cells was separated by sodium dodecyl sulfate–polyacrylamide gel electrophoresis (SDS-PAGE), followed by transferred onto nitrocellulose membranes (Merck, Darmstadt, Germany). The membranes were blocked with 2% skimmed milk (Anchor, Auckland, New Zealand) and incubated with primary antibodies overnight, followed by further incubation with species-matched horseradish peroxidase-conjugated secondary antibodies (Thermo Fisher Scientific, Waltham, MA, USA) for 1 h at room temperature. Immunoreactive protein bands were detected by incubation with Immobilon Western chemiluminescent reagents (Merck, Darmstadt, Germany), and the signals were captured by ChemiDoc XRS + (Bio-Rad, Hercules, CA, USA) and analyzed with Image Lab (Bio-Rad, Hercules, CA, USA). The following primary antibodies were used for Western blot: Goat anti-human IL-1RA (R&D Systems, Minneapolis, MN, USA), rabbit anti-human SOX2 (GeneTex, Hsinchu, Taiwan), rabbit anti-human EGF (GeneTex, Hsinchu, Taiwan), rabbit anti-human THY1 (Abcam, Waltham, MA, USA), rabbit anti-human c-KIT (Cell Signaling Technology, Danvers, MA, USA), rabbit anti-human p21 (GeneTex, Hsinchu, Taiwan), rabbit anti-human phospho-EGFR at Tyr1086 (GeneTex, Hsinchu, Taiwan), rabbit anti-human EGFR (GeneTex, Hsinchu, Taiwan), rabbit anti-human phospho-JNK1/2/3 at Thr183 (Abcam, Waltham, MA, USA), rabbit anti-human JNK1/2/3 (Abcam, Waltham, MA, USA), rabbit anti-human GAPDH (GeneTex, Hsinchu, Taiwan), and mouse anti-human β-actin (Sigma-Aldrich, St. Louis, MO, USA).

### Immunohistochemistry (IHC)

Formalin-fixed and paraffin-embedded normal and tumor tissue sections were immunostained using a Bond-Max automated IHC stainer (Leica Microsystems, Wetzlar, Germany) in accordance with the manufacturer’s instructions and our previous reports [24, 27]. The following primary antibodies were used for IHC: goat anti-human IL-1RA (R&D Systems, Minneapolis, MN, USA), rabbit anti-human SOX2 (GeneTex, Hsinchu, Taiwan), rabbit anti-human Ki67 (GeneTex, Hsinchu, Taiwan), rabbit anti-human phospho-EGFR at Tyr1086 (GeneTex, Hsinchu, Taiwan), and rabbit anti-human phospho-JNK1/2/3 at Thr183/Thr183/Thr221 (Abcam, Waltham, MA, USA). IHC images were captured using an Eclipse E600 microscope (Nikon, Tokyo, Japan), and total immunostaining scores for each tissue section were determined as the product of the percentage of positively stained cells (0, 0–4%; 1, 5–24%; 2, 25–49%; 3, 50–74%; 4, 75–100%) multiplied by the intensity of staining (0, negative; 1, weak; 2, moderate; 3, strong). To further analyze the association between IL-1RA expression and patient survival, the expression level of IL-1RA by IHC scoring was categorized into low versus high expression with cut-off values according to ROC curves [24, 27].

### Animal study

Experiments involving animals were approved by and in accordance with the guidelines and regulations of the Institutional Animal Care and Use Committee of Kaohsiung Medical University, Kaohsiung, Taiwan (Approval no. 108127). Three-week-old male NOD.CB17-*Prkdc*^*scid*^/JNarl mice were obtained from the National Laboratory Animal Center of Taiwan (https://www.nlac.narl.org.tw). For orthotopic xenografts, OECM-1 cells expressing luciferase (OECM-1-luc; 5 × 10^5^ cells per mouse) resuspended in 100 µL of phosphate-buffered saline were injected into mice through the intrabuccal route. At 1 week post-xenograft, the mice were randomly assigned to eight groups (five mice per group), including empty vector (EV) versus IL-1RA-OE from each of the untreated, metformin-treated, cisplatin-treated, and metformin combined with cisplatin-treated groups. The untreated groups were intraperitoneally injected with normal saline thrice per week. The metformin-treated group (200 mg/kg) was injected intraperitoneally three times per week. The cisplatin-treated group (2 mg/kg) was injected intraperitoneally once per week. In the metformin combined with cisplatin-treated groups, metformin (200 mg/kg) was administered three times per week and cisplatin (2 mg/kg) was administered once per week. All mice were monitored weekly using an IVIS Spectrum in vivo imaging system (PerkinElmer, Santa Clara, CA, USA), according to the manufacturer’s instructions and our previous report [24]. After treatment for 5 weeks, the mice were sacrificed, and orthotopic xenograft tumors were collected for tumor weight and volume measurements, followed by immunohistochemical analysis.

### Statistical analysis

Statistical analyses were performed using JMP version 10.0.1 for Windows (SAS Institute, Cary, NC, USA). The log-rank test was used to compare patient survival using Kaplan–Meier curve analysis. The Student’s t-test was used to compare the two groups. One-way analysis of variance (ANOVA) with post-hoc Tukey’s test was used for multiple group comparisons. Pearson correlation (r) was used to evaluate the correlation between the two protein expression levels by IHC. The data are presented as the mean ± SD from three independent experiments where applicable or as otherwise specified in individual figures. *P* values less than 0.05 were considered statistically significant.

## Results

### Clinical association of IL-1RA expression with OSCC progression and patient outcomes

**Fig. 1:**
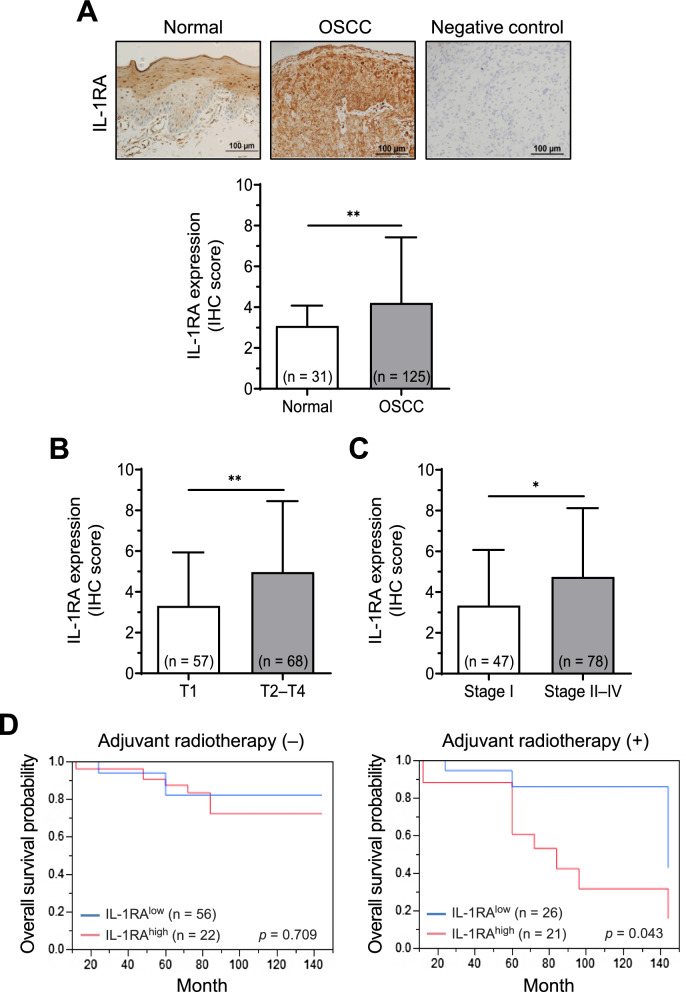
Association of IL-1RA expression with OSCC progression and patient outcomes. **A** Protein expression of IL-1RA in oral tumor tissues from OSCC patients (OSCC) and normal oral tissues from normal epithelial regions of fibroma patients (Normal) was analyzed by immunohistochemistry (IHC). The negative control for IL-1RA staining was performed in parallel with omission of primary antibodies. **B**–**C** Protein expression of IL-1RA in OSCC tumor tissues was analyzed by IHC for the comparison of primary tumor size (T1 versus T2-T4) in (**B**), and cancer stage (Stage I versus Stage II-IV) in (**C**). The scoring of IHC was described in the Methods section. Data were presented as mean ± SD with the indicated sample size in parentheses. *, *p* < 0.05; **, *p* < 0.01. **D** Overall survival probability was analyzed by Kaplan–Meier survival curves for patients with adjuvant radiotherapy ( +) and patients without adjuvant radiotherapy (−) according to the expression level of IL-1RA in OSCC tumor tissues (low versus high) as described in the Methods section. The sample size in each group was indicated in parentheses

To evaluate the association between IL-1RA expression and OSCC progression, immunohistochemical analysis was performed on OSCC tumor tissues and normal oral mucosal tissues adjacent to the oral fibroma tissues for comparison. The results indicated an elevated level of IL-1RA expression in OSCC tumor tissues compared to that in the normal oral mucosa (*p* = 0.001; Fig. 1A). Further analysis of the clinicopathological characteristics revealed that elevated IL-1RA expression in OSCC tumor tissues was associated with tumor size (*p* = 0.003; Fig. 1B) and cancer stage (*p* = 0.012; Fig. 1C). Moreover, there was an association between the expression of IL-1RA and the overall survival of patients with OSCC in response to treatment, in which high IL-1RA expression was associated with poorer overall survival probability in patients receiving adjuvant radiotherapy, but this association was not observed in patients who did not receive adjuvant radiotherapy (*p* = 0.043 and 0.709, respectively; Fig. 1D). These clinical data suggest that elevated IL-1RA expression is associated with adverse OSCC progression and poor treatment outcomes. Therefore, we performed gene knockdown and overexpression of IL-1RA in OSCC cells to investigate the impact of differential IL-1RA expression on cancer cell malignancy.

### IL-1RA promoted in vitro cancer cell growth and migration/invasion abilities via the mitochondrial metabolic pathway

Different levels of endogenous IL-1RA expression were detected in four human OSCC cell lines, including HSC-3, SAS, Ca9-22, and OECM-1, with HSC-3 and Ca9-22 cells showing higher IL-1RA expression than SAS and OECM-1 cells (Additional file 1: Figure S1A). A screen for knockdown clones of IL-1RA revealed better efficacy of shIL-1RA#1 and #2 (Additional file 1: Figure S1B). Therefore, in subsequent studies, HSC-3 and Ca9-22 cells were selected for gene knockdown of IL-1RA by shIL-1RA#1 and #2 and OECM-1 cells for gene overexpression of full-length IL-1RA (Additional file 1: Figure S1C).

**Fig. 2:**
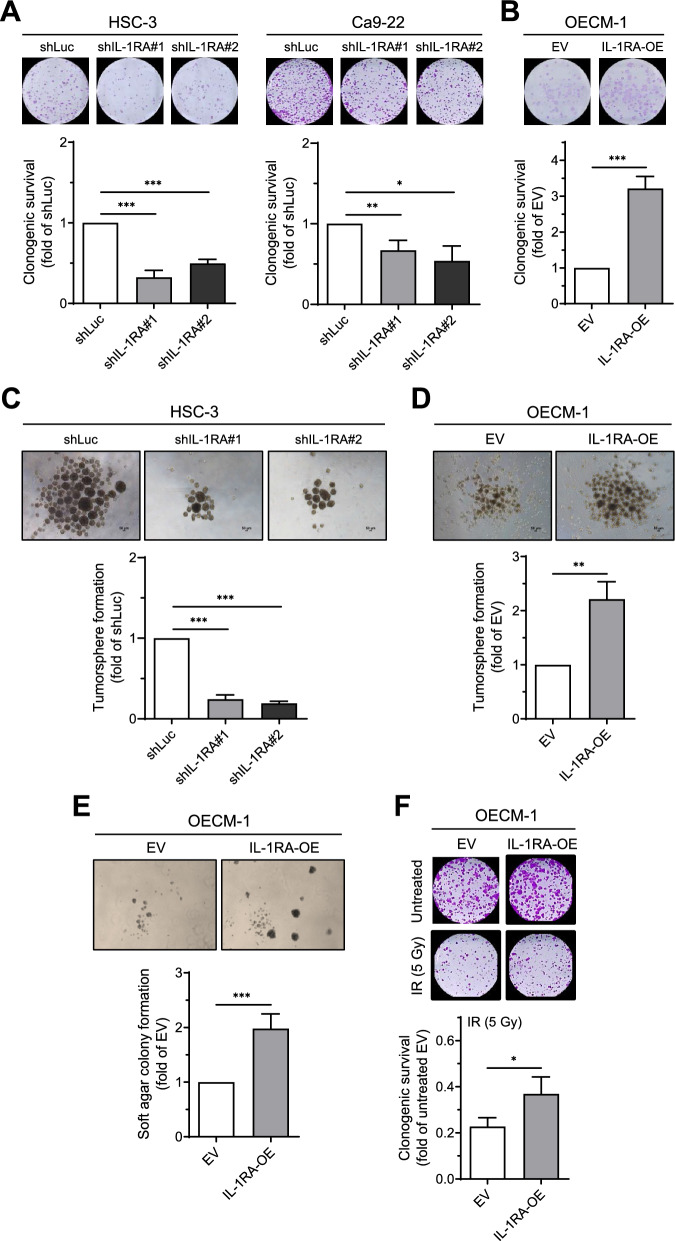
Effect of IL-1RA expression on in vitro cell growth of OSCC cells. **A**–**F** Following knockdown of IL-1RA expression in HSC-3 and Ca9-22 cells and overexpression of IL-1RA in OECM-1 cells, assessments were performed for clonogenic survival (**A**, **B**), tumorsphere formation (**C**, **D**), and colony formation in soft agar (**E**). In (**F**), clonogenic survival was assessed in OECM-1 cells with overexpression of IL-1RA and control cells in the presence or absence of ionizing radiation treatment. Data were presented as mean ± SD from three independent experiments. *, *p* < 0.05; **, *p* < 0.01; ***, *p* < 0.001. shLuc, knockdown of firefly luciferase; shIL-1RA, knockdown of IL-1RA; EV, empty vector; IL-1RA-OE, overexpression of IL-1RA

Colony forming assay and tumorsphere formation assay have been used for the determination of cancer cell stemness [28, 29]. Using a clonogenic survival assay, we observed that knockdown of IL-1RA in HSC-3 and Ca9-22 cells reduced cancer cell growth (Fig. 2A), whereas overexpression of IL-1RA in OECM-1 cells increased cancer cell growth (Fig. 2B). Furthermore, knockdown of IL-1RA in HSC-3 cells reduced tumorsphere formation (Fig. 2C), whereas overexpression of IL-1RA in OECM-1 cells increased tumorsphere formation (Fig. 2D). In addition, overexpression of IL-1RA in OECM-1 cells enhanced colony formation on soft agar (Fig. 2E). Analysis of the cell cycle distribution revealed that knockdown of IL-1RA in HSC-3 cells led to increased G0/G1 phase and decreased G2/M phase (Additional file 1: Figure S2A), whereas overexpression of IL-1RA in OECM-1 cells had the opposite effect on cell cycle distribution (Additional file 1: Figure S2B). Concordantly, the expression of p21 was increased in HSC-3 cells with knockdown IL-1RA but decreased in OECM-1 cells overexpressing IL-1RA (Additional file 1: Figure S2C and D). To examine the effect of IL-1RA expression on cancer cell growth under the influence of ionizing radiation (IR), OECM-1 cells were treated with IR followed by a clonogenic survival assay. We found that while ionizing irradiation caused a significant reduction in cell growth in both IL-1RA overexpression and control EV groups, the IL-1RA overexpression group had more surviving cells after IR treatment than the EV controls (Fig. 2F). These data indicate that alteration of IL-1RA expression affects cancer cell growth in OSCC cells, including clonogenic survival and colony formation in soft agar, and tumorsphere formation, which represent the properties of cancer stemness-associated growth [30–32].

**Fig. 3:**
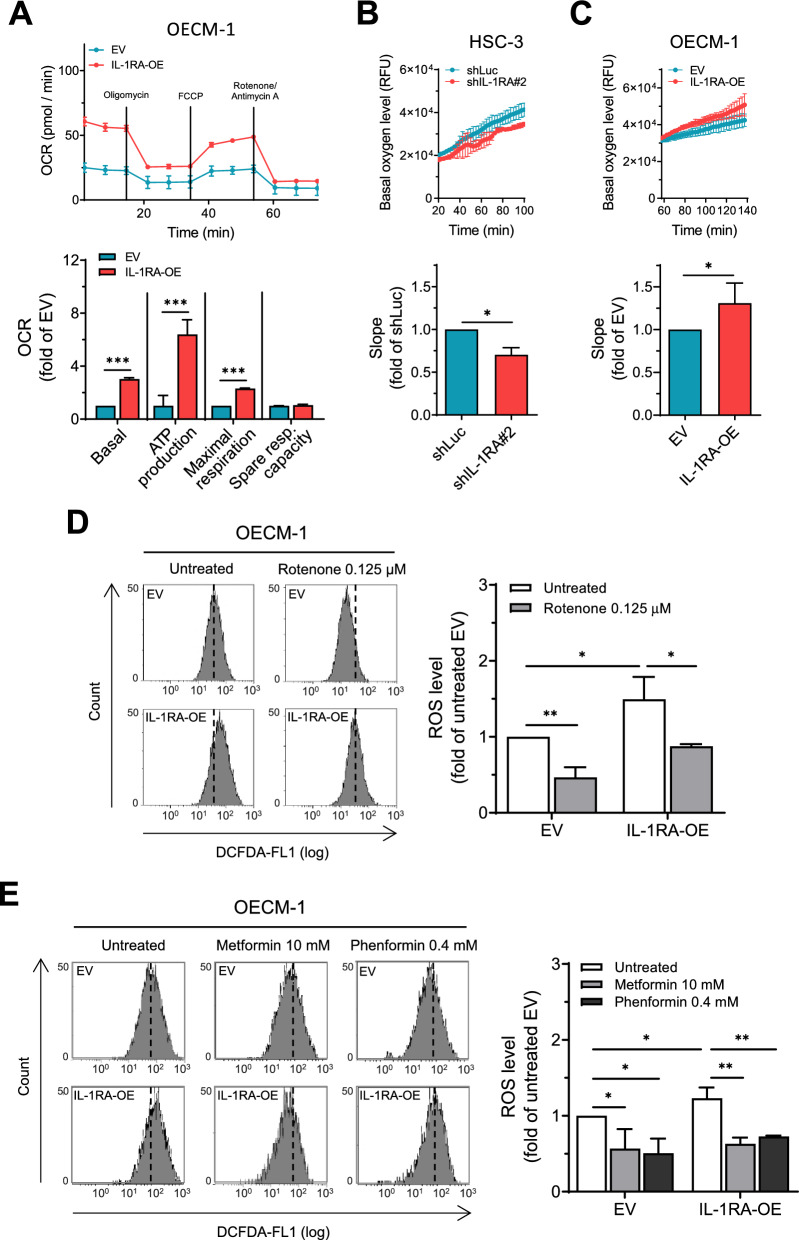
Effect of IL-1RA expression on mitochondrial metabolism and reactive oxygen species (ROS) formation in OSCC cells. **A** Multiple status of mitochondrial metabolism was measured by oxygen consumption rate (OCR) using Agilent Seahorse XFe24 Analyzer via sequential delivery of the indicated mitochondrial modulators (oligomycin, FCCP, and a mixture of rotenone and antimycin A) to OECM-1 cells with overexpression of IL-1RA and control cells. **B**–**C** Basal OCR indicated by the slope (∆RFU/∆min) was measured with CLARIOstar Plus multi-mode plate reader using a commercial oxygen consumption-labeling kit, for HSC-3 cells with knockdown of IL-1RA and control cells in (**B**), and OECM-1 cells with overexpression of IL-1RA and control cells in (**C**). **D**–**E** ROS formation was measured by flow cytometry after DCFDA labeling of OECM-1 cells with overexpression of IL-1RA and control cells in the presence or absence of rotenone in (**D**), and metformin or phenformin in (**E**). Data were presented as mean ± SD from three independent experiments. *, *p* < 0.05; **, *p* < 0.01; ***, *p* < 0.001. RFU, relative fluorescence unit; shLuc, knockdown of firefly luciferase; shIL-1RA, knockdown of IL-1RA; EV, empty vector; IL-1RA-OE, overexpression of IL-1RA

**Fig. 4:**
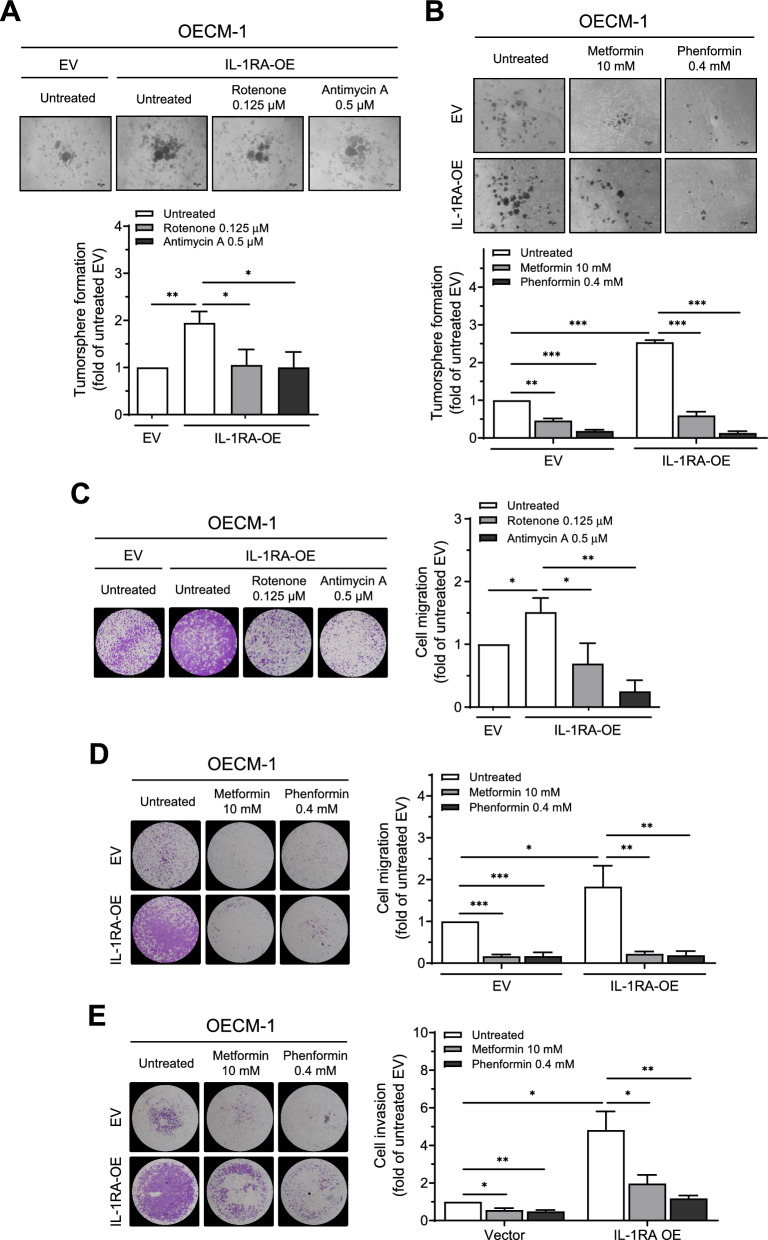
Involvement of mitochondrial metabolism in IL-1RA-promoted malignant behaviors of OSCC cells. **A**–**B** OECM-1 cells with IL-1RA overexpression and control cells were assessed for tumorsphere formation in the presence or absence of rotenone or antimycin A in (**A**), and metformin or phenformin in (**B**). **C**–**D** OECM-1 cells with IL-1RA overexpression and control cells were assessed for transwell cell migration ability in the presence or absence of rotenone or antimycin A in (**C**), and metformin or phenformin in (**D**). **E** OECM-1 cells with IL-1RA overexpression and control cells were assessed for transwell cell invasion ability in the presence or absence of metformin or phenformin. Data were presented as mean ± SD from three independent experiments. *, *p* < 0.05; **, *p* < 0.01; ***, *p* < 0.001. EV, empty vector; IL-1RA-OE, overexpression of IL-1RA

Mitochondrial metabolism has been implicated in oncogenesis, such as the acquisition of cancer stemness properties, metastatic ability, and radioresistance [7, 9, 33]. In this study, we demonstrated that IL-1RA positively correlated with cancer cell stemness (Fig. 2). Therefore, we further examined the role of mitochondrial metabolism in IL-1RA-associated oral cancer cell malignancy. As shown in Fig. 3A, overexpression of IL-1RA in OECM-1 cells increased the oxygen consumption rate (OCR), an indicator of mitochondrial oxidative phosphorylation [34, 35], as indicated by the upregulated basal OCR, ATP production, and maximal respiration using the Seahorse Analyzer. In concordance with this result, altered basal OCR was observed in HSC-3 cells with knockdown of IL-1RA and in OECM-1 cells overexpressing IL-1RA using a different apparatus (Fig. 3B and C). In contrast, Additional file 1: Figure S3A and B show that the effect of differential IL-1RA expression on the extracellular acidification rate (ECAR; an indicator of glycolytic level [34, 35] was the opposite compared to the results of OCR in Fig. 3B and C. Levels of reactive oxygen species (ROS) were increased by overexpression of IL-1RA in OECM-1 cells and were reduced in the presence of the mitochondrial complex inhibitor rotenone (Fig. 3D), metformin, and phenformin (Fig. 3E) [36, 37]. Furthermore, application of mitochondrial complex inhibitors to OECM-1 cells overexpressing IL-1RA effectively reduced IL-1RA-promoted tumorsphere formation (Fig. 4A and B). IL-1RA-promoted cell migration (Fig. 4C, D, and Additional file 1: Figure S4A) and invasion (Fig. 4E) in OECM-1 cells were also reduced by mitochondrial complex inhibitors under circumstances where these inhibitors did not affect the viability of OECM-1 cells within the same time window of treatment (Additional file 1: Figure S4B–D). Altogether, these data suggest that IL-1RA-promoted OSCC malignant behaviors, such as tumorspheric growth and cell migration/invasion abilities, are mediated through the mitochondrial metabolic pathway.

### Involvement of mitochondrial metabolism in IL-1RA-associated SOX2 expression

**Fig. 5:**
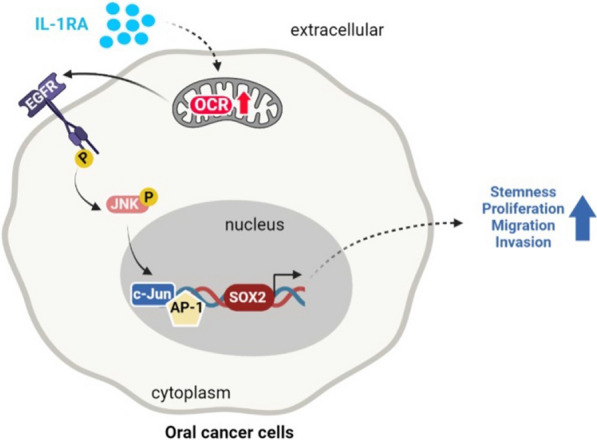
Involvement of mitochondrial metabolism in the expression of IL-1RA-associated cancer stemness markers. **A** PCR array was applied to screen a set of cancer stem cell-related genes in HSC-3 cells with knockdown of IL-1RA, in which the expression of each gene was quantitated relative to that in control cells (dashed line). **B**–**C** Four candidates (SOX2, THY1, EGF, and KIT), as indicated by the solid rectangle in (**A**), were further examined by Western blot for their protein expression in HSC-3 and Ca9-22 cells with knockdown of IL-1RA in (**B**), and OECM-1 cells with overexpression of IL-1RA in (**C**), along with the control cells. **D** Protein expression of SOX2 in OECM-1 cells with overexpression of IL-1RA and control cells was examined by Western blot in the presence or absence of rotenone or antimycin A. Data were presented as mean ± SD from three independent experiments in (**B**–**D**). *, *p* < 0.05; **, *p* < 0.01; ***, *p* < 0.001. Rot., rotenone; Ant. A, antimycin A; shLuc, knockdown of firefly luciferase; shIL-1RA, knockdown of IL-1RA; EV, empty vector; IL-1RA-OE, overexpression of IL-1RA

To investigate the potential involvement of cancer stemness-related genes in IL-1RA-promoted OSCC malignancy, a screening approach using a PCR array was applied, which showed that four stemness markers, including *SOX2*, *THY1*, *EGF*, and *KIT*, were most downregulated in HSC-3 cells with IL-1RA knockdown (Fig. 5A and Additional file 1: Figure S5). The protein expression levels of these four candidates were further examined by western blotting, which showed that SOX2 was consistently downregulated in HSC-3 and Ca9-22 cells with IL-1RA knockdown (Fig. 5B) and upregulated in OECM-1 cells overexpressing IL-1RA (Fig. 5C). Moreover, IL-1RA-promoted SOX2 expression in OECM-1 cells was reduced in the presence of mitochondrial complex inhibitors (Fig. 5D), suggesting that the effect of IL-1RA on SOX2 expression is mediated through the mitochondrial metabolic pathway.

### IL-1RA promoted SOX2 expression via phosphorylation of EGFR and JNK downstream of mitochondrial metabolic pathway

**Fig. 6:**
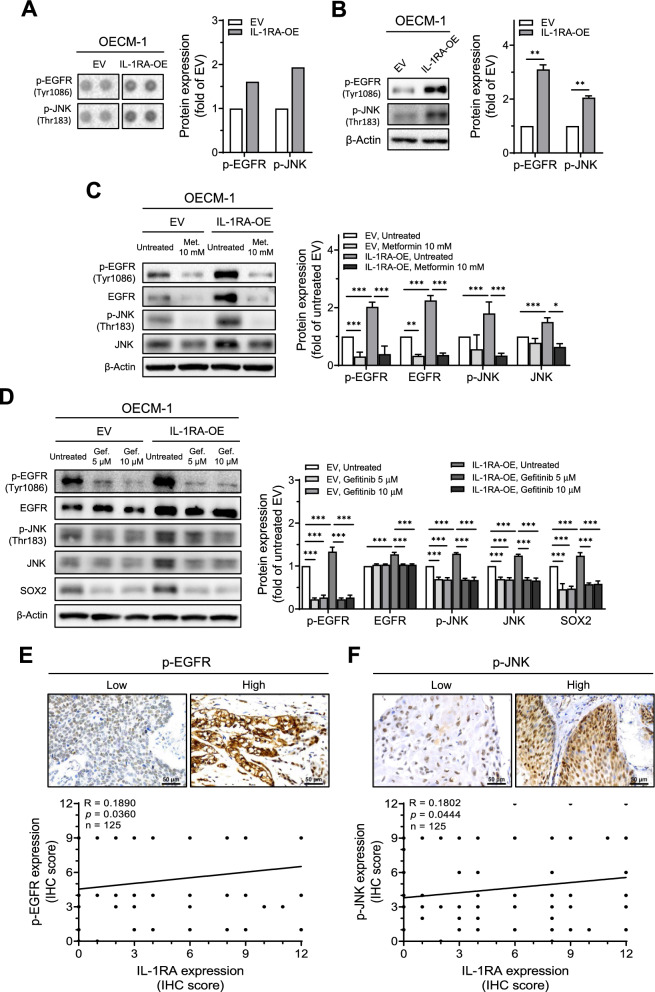
Involvement of mitochondrial metabolism in IL-1RA-associated EGFR/JNK activation and SOX2 expression. **A**–**B** Up-regulated phosphorylation of EGFR and JNK was shown by phospho-kinase proteome array screening for the phosphorylation status of a set of kinases, with duplicate detection for each kinase, in OECM-1 cells with overexpression of IL-1RA in (**A**), which was further confirmed by Western blot in (**B**). **C** Protein expression of phosphorylated EGFR and JNK, along with their unphosphorylated forms, in OECM-1 cells with overexpression of IL-1RA and control cells was examined by Western blot in the presence or absence of metformin. **D** Protein expression of phosphorylated EGFR and JNK, along with their unphosphorylated forms, and SOX2 in OECM-1 cells with overexpression of IL-1RA and control cells was examined by Western blot in the presence or absence of gefitinib. Data were presented as mean ± SD from three independent experiments in (**B**–**D**). *, *p* < 0.05; **, *p* < 0.01; ***, *p* < 0.001. **E**–**F** Correlation between IL-1RA expression and phosphorylation of EGFR (**E**) and JNK (**F**) in OSCC tumor tissues from patients was analyzed according to IHC scoring, and was evaluated by Pearson correlation (r). Met., metformin; Gef., gefitinib; p-EGFR, phosphorylated EGFR; p-JNK, phosphorylated JNK; EV, empty vector; IL-1RA-OE, overexpression of IL-1RA

To further investigate the signaling molecules involved in IL-1RA-associated OSCC malignancy, an antibody array was used to screen for the phosphorylation status of kinases affected by IL-1RA. Antibody array screening showed that the expression levels of phosphorylated EGFR (p-EGFR) and phosphorylated JNK (p-JNK) were increased in OECM-1 cells overexpressing IL-1RA (Fig. 6A), which was confirmed by western blotting (Fig. 6B). The increase in p-EGFR and p-JNK induced by IL-1RA overexpression in OECM-1 cells was reduced in the presence of metformin, along with a reduction in EGFR and JNK expression (Fig. 6C). We also found that inhibition of EGFR by gefitinib (an EGFR inhibitor [38]) reduced the expression of p-JNK and SOX2 (Fig. 6D). In addition, a correlation between IL-1RA expression and the expression of p-EGFR (Fig. 6E) and p-JNK (Fig. 6F) was observed in oral tumor tissues from patients with OSCC. Taken together, these data suggest that IL-1RA-associated OSCC malignancy is regulated via EGFR-mediated JNK signaling and SOX2 expression, which lie downstream of the mitochondrial metabolic pathway.

### IL-1RA-promoted in vivo tumor growth and p-EGFR/p-JNK/SOX2 expression were suppressed by metformin and cisplatin

**Fig. 7:**
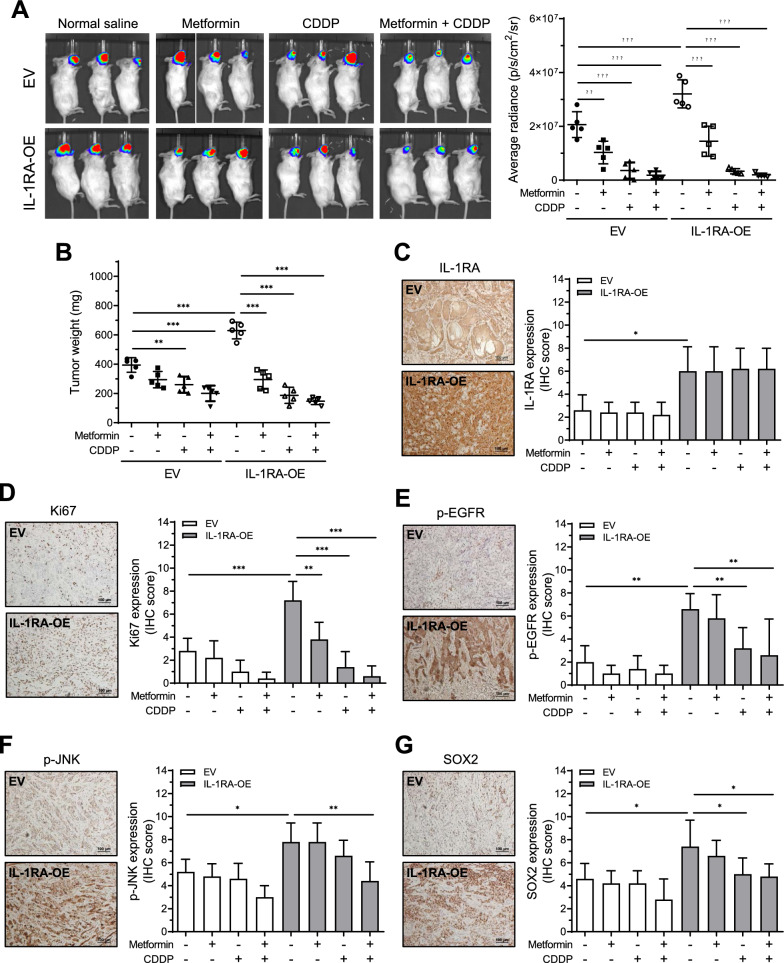
Effect of IL-1RA expression on in vivo tumor growth of OSCC in xenograft mice and the therapeutic potential of metformin. **A** Orthotopic xenograft of OECM-1 cells with overexpression of IL-1RA or control cells in mice was measured weekly by in vivo imaging system (IVIS) for the groups treated with or without metformin and/or CDDP. The data shown in (**A**) were the measurements at endpoint of the experiment before sacrifice of the mice. **B** After sacrifice of the mice at endpoint of the experiment, the xenograft tumors were collected and tumor weight was measured. **C**–**G** After sacrifice of the mice at endpoint of the experiment, the xenograft tumors were collected and analyzed for protein expression of IL-1RA (**C**), Ki67 (**D**), phosphorylated EGFR (**E**), phosphorylated JNK (**F**), and SOX2 (**G**). Data were presented as mean ± SD from five mice in each group. *, *p* < 0.05; **, *p* < 0.01; ***, *p* < 0.001. CDDP, cisplatin; p-EGFR, phosphorylated EGFR; p-JNK, phosphorylated JNK; EV, empty vector; IL-1RA-OE, overexpression of IL-1RA

The effect of IL-1RA on in vivo tumor growth was evaluated in mice via orthotopic xenografts of OECM-1-luc cells with IL-1RA overexpression or empty vector as a control (Additional file 1: Figure S6A), and the effect of treatment with metformin, cisplatin (also known as CDDP; a chemotherapeutic agent [39]), or a combination of both, was further evaluated (Additional file 1: Figure S6B). Tumor formation was measured weekly using an in vivo imaging system (IVIS). At the end of the experiment, a higher level of bioluminescence resulting from luciferase activity in the orthotopic xenograft area was observed in mice treated with IL-1RA-overexpressing OECM-1-luc cells (Fig. 7A). Furthermore, the mice treated with metformin, CDDP, or a combination of both showed reduced levels of bioluminescence (Fig. 7A), as well as reduced tumor weight (Fig. 7B) and tumor volume (Additional file 1: Figure S6C and D), in which the combined metformin and CDDP treatment exhibited the greatest effect on the inhibition of tumor formation. Immunohistochemical analysis for the expression of IL-1RA, Ki-67 (a cancer proliferation marker [40]), p-EGFR, p-JNK, and SOX2 revealed that IL-1RA expression remained high in the xenograft tumors collected from the mice groups with IL-1RA-overexpressing OECM-1-luc cells (Fig. 7C), whereas the elevated expression of IL-1RA-associated Ki-67, p-EGFR, p-JNK, and SOX2 was reduced in the groups treated with metformin, CDDP, or a combination of both (Fig. 7D–G). These data suggested that IL-1RA promoted in vivo tumor growth with increased p-EGFR, p-JNK, and SOX2 expression, which was suppressed by treatment with metformin or CDDP. In addition, combined treatment with metformin and CDDP may have a greater biological effect than treatment with metformin or CDDP.

## Discussion

**Fig. 8:**
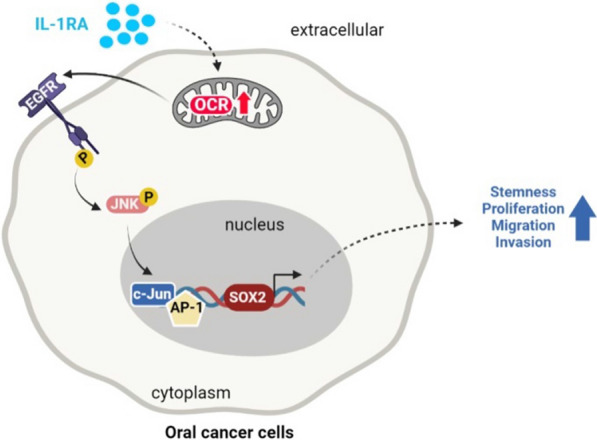
Schematic summary for the current study. The results of the current study suggest that mitochondrial metabolism may be crucial in mediating the IL-1RA-promoted malignant behaviors of OSCC cells, such as cancer stemness-associated tumor growth and migration/invasion abilities, through a pathway involving EGFR/JNK activation and SOX2 expression

IL-1RA, a critical regulator of the IL-1 signaling network, has emerged as a key player in cancer development [41, 42], yet its role in oral cancer malignancy remains to be elucidated. In this study, we highlighted the significance of IL-1RA as a clinical marker of OSCC and explored its biological mechanisms and therapeutic potential. Specifically, our data suggest that elevated IL-1RA levels are associated with adverse OSCC progression and poor patient outcomes. In addition, IL-1RA-promoted OSCC malignancy involved enhanced properties of cancer stemness via a mitochondrial metabolism-associated EGFR/JNK pathway, which may be suppressed by application of mitochondrial complex inhibitors both in vitro and in vivo (Fig. 8).

### A novel IL-1RA/EGFR/JNK pathway in oral cancer

EGFR is a transmembrane protein that can transduce signals via translocation to intracellular organelles upon stimulation of various cancer cells, in addition to its canonical receptor tyrosine kinase activity [43–45]. For example, mitochondrial translocation of EGFR occurs in breast cancer cells in response to EGF stimulation, which subsequently induces the phosphorylation of cytochrome c oxidase subunit II (Cox II) in the mitochondria and enhances cancer cell survival [46, 47]. Mitochondrial translocation of EGFR following EGF stimulation was also observed in non-small cell lung cancer (NSCLC), where increased mitochondrial EGFR upregulates ATP production and enhances the metastatic ability of NSCLC cells [48]. Another study revealed that nuclear translocation of EGFR is regulated by AKT, which is associated with gefitinib resistance in breast cancer cells through enhanced gene expression of the breast cancer-resistant protein (*BCRP*; also known as ATP-binding cassette subfamily G member 2, *ABCG2*) [49]. Furthermore, attenuation of estrogen receptor β expression leads to increased EGFR nuclear translocation, which is associated with poor patient outcomes in prostate cancer [50]. Our current study revealed for the first time that the activation of EGFR by IL-1RA induced the activation of the JNK pathway and was associated with enhanced malignant behavior of OSCC cells. Clinicopathological analysis supported these findings by revealing a positive correlation between the expression of IL-1RA and phosphorylated EGFR and JNK in OSCC tumor tissues. This newly identified pathway involving IL-1RA/EGFR/JNK indicates that targeting the EGFR pathway may be a potential therapeutic strategy for IL-1RA-associated OSCC malignancy. Other than our study, it has also been reported that miR-100 promotes tumor metastasis in mouse breast cancer via the STAT5a/IL-1RA signaling pathway [51]. On the other hand, the anti-cancer activity of IL-1RA has been reported, with a previous study showing that IL-1RA suppresses esophageal cancer cell growth through inhibition of IL-1α-VEGF signaling [52].

Along with our findings, KRAS has also been found to be associated with cell invasion, tumorigenesis, and lymph node metastasis in oral cancer [53]. Wang et al. demonstrated that KRAS is a novel prognostic marker in OSCC among Taiwanese people [54]. Oncogenic RAS targets metabolic reprogramming in various cancer types, including pancreatic cancer, lung cancer, colorectal cancer, and head and neck cancer [55]. Interestingly, combinational treatment of 5-florouracil and IL-1RA decreases the proliferation ability in colon cancer cells with KRAS mutation [56]. Although the cross-talk between IL-1RA and KRAS in oral cancer remains unclear, the underlying mechanism deserves further exploration.

### OSCC cancer stemness is enhanced by IL-1RA

Cancer stemness, the enhanced ability for self-proliferation and cancer cell motility, has been shown to correlate with tumor recurrence and metastasis and is a critical area in oral cancer research [8, 57, 58]. Our current study showed that IL-1RA enhanced cancer stemness-associated properties of OSCC cells, such as self-proliferation, anchorage-independent growth, and cell migration/invasion abilities. In addition, IL-1RA promoted the expression of SOX2 in OSCC cells. As SOX2 is a pleiotropic transcription factor and an important hallmark of cancer stemness, with functional roles in regulating tumor growth and metastasis [59, 60], our current findings suggest that increased SOX2 expression may play a role in IL-1RA-promoted OSCC malignancy.

### Inhibition of mitochondrial metabolism may abrogate IL-1RA-mediated malignant behaviors

Alteration of cellular energy and redox status via mitochondrial metabolism, a process known as mitochondrial metabolic reprogramming, has been shown to be associated with cancer progression [61, 62]. As a result, targeting mitochondria by inhibiting mitochondrial complexes presents a novel therapeutic strategy for cancer [63, 64]. Our in vitro data showed that IL-1RA overexpression increased the rate of mitochondrial oxidative metabolism and inhibition of mitochondrial electron transfer complexes, including the use of conventional compound inhibitors (e.g., metformin, phenformin, rotenone, or antimycin A [39, 65]) and novel peptide inhibitors (e.g., CT20 [66] or MITOx20 [67]), suppressed IL-1RA-promoted malignant behaviors of OSCC cells. In particular, the widely used diabetic medication metformin inhibits mitochondrial complex 1 to reduce tumorigenesis, with cancer risk reduction noted in epidemiological studies [68–70]. On the other hand, Cisplatin is a commonly used chemotherapeutic agent for treatment of oral cancer [71, 72]. The current study explored the potential of metformin as a therapeutic drug for IL-1RA-associated OSCC malignancy in an orthotopic xenograft mouse model, showing that IL-1RA-promoted tumor growth and activation of EGFR/JNK and SOX2 expression in tumor tissues was reduced by metformin or cisplatin treatment, and the inhibitory effect was further augmented by combination therapy. Together, these data not only suggest a crucial involvement of mitochondrial metabolism in IL-1RA-promoted OSCC growth with participation of the EGFR/JNK/SOX2 pathway but also provide pre-clinical evidence for drug repurposing of metformin as an adjunctive treatment in combination with existing cancer therapies to enhance the therapeutic response [73, 74]. Furthermore, our data may help identify those who would benefit most from such adjunctive treatments, suggesting risk stratification of patients with high IL 1RA tumor tissue levels requiring radiotherapy to additional metformin treatment.

### Limitations and future work

While this study has unveiled a novel mechanism underlying IL-1RA-promoted OSCC malignancy through mitochondrial metabolism-associated EGFR/JNK activation and SOX2 expression, there are some limitations inherent in our current data, as well as areas that warrant further investigation. A limitation of this study is that the clinical samples used in this study were all from the decoded biological database and therefore we were unable to study the effect of other underlying conditions on the expression of IL-1RA. Also, whether IL-1RA-activated EGFR transduces signals via its receptor tyrosine kinase activity or via translocation to intracellular organelles remains to be determined. The genetic control of SOX2 expression induced by IL-1RA in OSCC cells also requires further investigation, such as the potential for transcriptional regulation by c-Jun, which can bind to the promoter region of SOX2 [75, 76]. Furthermore, apart from the effect of metformin on in vivo tumor growth examined in this study, the therapeutic potential of metformin on metastasis in a pre-clinical in vivo model merits further investigation.

## Conclusions

The current study demonstrates a critical role for IL-1RA in the pathogenesis of OSCC, and its elevated expression in OSCC tumor tissues may be considered an adverse clinical marker for cancer progression and treatment response. In addition, the newly identified biological mechanisms linking IL-1RA and OSCC malignancy suggest that mitochondrial metabolism plays a crucial role in regulating the malignant behavior of OSCC cells. In particular, the administration of metformin, which has been proposed as a repurposed drug for cancer treatment [68, 69, 73, 74], alleviates IL-1RA-promoted OSCC malignancy through EGFR/JNK signaling and SOX2 expression. These findings not only reveal the impact of IL-1RA and its associated mechanisms on OSCC malignancy but also provide insights for further development of targeted therapies, such as the potential for targeted delivery of metformin to mitochondria [77] or cancer cell surfaces [78], as a treatment strategy for OSCC.

## Supplementary Information

**Additional file 1: Figure S1. A** Endogenous protein expression of IL-1RA was examined by Western blot in four human OSCC cell lines, including HSC-3, SAS, Ca9-22, and OECM-1 cells, along with human dysplastic oral keratinocyte (DOK) cells for comparison. Data were presented as mean ± SD from three independent experiments, and the number indicates fold of protein expression in each cell line relative to DOK cells. **B** Representative results by Western blot showed the knockdown efficiency from four clones of shRNA targeting different consensus regions of human *IL1RN* (Accession: NM_000577). The number indicates fold of protein expression in HSC-3 cells carrying individual shRNA clone relative to the control cells. **C** Representative results by Western blot showed the protein expression of knockdown of IL-1RA in HSC-3 or Ca9-22 cells using shRNA clones #1 and #2 that had a better knockdown efficiency compared to shRNA clones #3 and #4 in (**B**), and the protein expression of overexpression of IL-1RA in OECM-1 cells carrying a vector that expresses full-length human *IL1RN* (Accession: NM_173841.2). GAPDH, glyceraldehyde-3-phosphate dehydrogenase; shLuc, knockdown of firefly luciferase; shIL-1RA, knockdown of IL-1RA; EV, empty vector; IL-1RA-OE, overexpression of IL-1RA. **Figure S2. A-B** Cell cycle distribution was analyzed by flow cytometry in HSC-3 cells with knockdown of IL-1RA in (**A**), and OECM-1 cells with overexpression of IL-1RA in (**B**), along with the control cells. **C-D** Protein expression of p21 was examined by Western blot in HSC-3 cells with knockdown of IL-1RA in (**C**), and OECM-1 cells with overexpression of IL-1RA in (**D**), along with the control cells. Data were presented as mean ± SD from three independent experiments. *, *p* < 0.05; **, *p* < 0.01. shLuc, knockdown of firefly luciferase; shIL-1RA, knockdown of IL-1RA; EV, empty vector; IL-1RA-OE, overexpression of IL-1RA. **Figure S3. A-B** Basal extracellular acidification rate (ECAR) indicated by the slope (∆RFU/∆min) was measured in CLARIOstar Plus multi-mode plate reader using a commercial glycolytic flux-labeling kit for HSC-3 cells with knockdown of IL-1RA in (**A**), and OECM-1 cells with overexpression of IL-1RA in (**B**), along with the control cells. Data were presented as mean ± SD from three independent experiments. *, *p* < 0.05; **, *p* < 0.01. shLuc, knockdown of firefly luciferase; shIL-1RA, knockdown of IL-1RA; EV, empty vector; IL-1RA-OE, overexpression of IL-1RA. **Figure S4. A** Overexpression of IL-1RA in OECM-1 cells and the control cells were assessed for transwell cell migration ability in the presence or absence of CT20 or MITOx20. **B-C** Overexpression of IL-1RA in OECM-1 cells and the control cells were assessed for cell viability by XTT assay in the presence or absence of rotenone or antimycin A in (**B**), metformin or phenformin in (**C**), and CT20 or MITOx20 in (**D**). Data were presented as mean ± SD from three independent experiments. **, *p* < 0.01. EV, empty vector; IL-1RA-OE, overexpression of IL-1RA. **Figure S5.** List of the genes screened by PCR array as shown in Fig. 5A. **Figure S6. A** Representative results by Western blot showed the protein expression of overexpression of IL-1RA in OECM-1-luc cells carrying a vector that expresses full-length human *IL1RN* (Accession: NM_173841.2). **B** Schematic summary of the animal study. **C** Photograph of xenograft tumors collected from each group at endpoint of the experiment. **D** Xenograft tumors collected at endpoint of the experiment were measured for tumor volumes. CDDP, cisplatin; EV, empty vector; IL-1RA-OE, overexpression of IL-1RA.

## Data Availability

The datasets used and/or analyzed during the current study are available from the corresponding author upon reasonable request.

## Abbreviations

ANOVA: One-way analysis of variance
ECAR: Extracellular acidification rate
EGF: Epidermal growth factor
EGFR: Epidermal growth factor receptor
EV: Empty vector
FBS: Fetal bovine serum
FGF-basic: Fibroblast growth factor-basic
FCCP: Carbonyl cyanide 4-(trifluoromethoxy)phenylhydrazone
GAPDH: Glyceraldehyde-3-phosphate dehydrogenas
IHC: Immunohistochemistry
IL-1: Interleukin-1
IL-1RA: Interleukin-1 receptor antagonist
IL-1RA-OE: Overexpression of IL-1RA
IR: Ionizing radiation
IVIS: In vivo imaging system
JNK: C-Jun N-terminal kinase
OCR: Oxygen consumption rate
OSCC: Oral squamous cell carcinoma
PCR: Polymerase chain reaction
RFU: Relative fluorescence unit
ROS: Reactive oxygen species
SDS-PAGE: Sodium dodecyl sulfate–polyacrylamide gel electrophoresis
shIL-1RA: Knockdown of IL-1RA
shLuc: Knockdown of firefly luciferase
SOX2: SRY-box transcription factor 2
