# Supplementary material for: IL-1RA promotes oral squamous cell carcinoma malignancy through mitochondrial metabolism-mediated EGFR/JNK/SOX2 pathway
Source: J Transl Med. 2023 Jul 17;21:473. doi: 10.1186/s12967-023-04343-9 (PMC10351194; doi:10.1186/s12967-023-04343-9)
Supplement: Supplementary file 1 — Additional file 1: Figure S1. A Endogenous protein expression of IL-1RA was examined by Western blot in four human OSCC cell lines, including HSC-3, SAS, Ca9-22, and OECM-1 cells, along with human dysplastic oral keratinocyte (DOK) cells for comparison. Data were presented as mean ± SD from three independent experiments, and the number indicates fold of protein expression in each cell line relative to DOK cells. B Representative results by Western blot showed the knockdown efficiency from four clones of shRNA targeting different consensus regions of human IL1RN (Accession: NM_000577). The number indicates fold of protein expression in HSC-3 cells carrying individual shRNA clone relative to the control cells. C Representative results by Western blot showed the protein expression of knockdown of IL-1RA in HSC-3 or Ca9-22 cells using shRNA clones #1 and #2 that had a better knockdown efficiency compared to shRNA clones #3 and #4 in (B), and the protein expression of overexpression of IL-1RA in OECM-1 cells carrying a vector that expresses full-length human IL1RN (Accession: NM_173841.2). GAPDH, glyceraldehyde-3-phosphate dehydrogenase; shLuc, knockdown of firefly luciferase; shIL-1RA, knockdown of IL-1RA; EV, empty vector; IL-1RA-OE, overexpression of IL-1RA. Figure S2. A-B Cell cycle distribution was analyzed by flow cytometry in HSC-3 cells with knockdown of IL-1RA in (A), and OECM-1 cells with overexpression of IL-1RA in (B), along with the control cells. C-D Protein expression of p21 was examined by Western blot in HSC-3 cells with knockdown of IL-1RA in (C), and OECM-1 cells with overexpression of IL-1RA in (D), along with the control cells. Data were presented as mean ± SD from three independent experiments. *, p < 0.05; **, p < 0.01. shLuc, knockdown of firefly luciferase; shIL-1RA, knockdown of IL-1RA; EV, empty vector; IL-1RA-OE, overexpression of IL-1RA. Figure S3. A-B Basal extracellular acidification rate (ECAR) indicated by the slope (∆RFU/∆min) was measur [file 12967_2023_4343_MOESM1_ESM.pptx]

## Slide 1
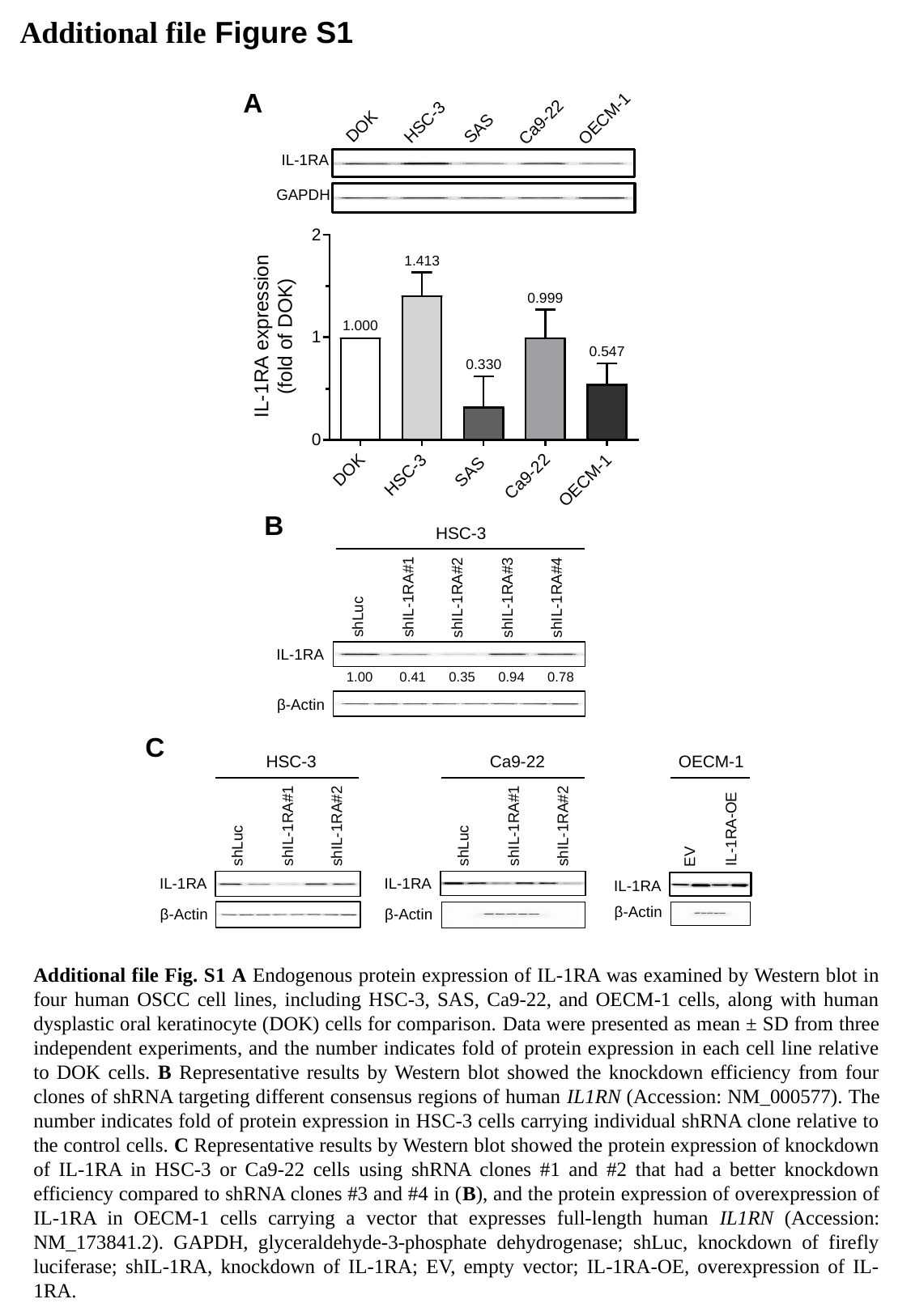

Additional file Figure S1
A
OECM-1
Ca9-22
HSC-3
DOK
SAS
IL-1RA
GAPDH
B
HSC-3
shIL-1RA#1
shIL-1RA#3
shIL-1RA#4
shIL-1RA#2
shLuc
IL-1RA
 1.00 0.41 0.35 0.94 0.78
β-Actin
C
OECM-1
HSC-3
Ca9-22
shIL-1RA#1
shIL-1RA#1
IL-1RA-OE
shIL-1RA#2
shIL-1RA#2
shLuc
shLuc
EV
IL-1RA
IL-1RA
IL-1RA
β-Actin
β-Actin
β-Actin
Additional file Fig. S1 A Endogenous protein expression of IL-1RA was examined by Western blot in four human OSCC cell lines, including HSC-3, SAS, Ca9-22, and OECM-1 cells, along with human dysplastic oral keratinocyte (DOK) cells for comparison. Data were presented as mean ± SD from three independent experiments, and the number indicates fold of protein expression in each cell line relative to DOK cells. B Representative results by Western blot showed the knockdown efficiency from four clones of shRNA targeting different consensus regions of human IL1RN (Accession: NM_000577). The number indicates fold of protein expression in HSC-3 cells carrying individual shRNA clone relative to the control cells. C Representative results by Western blot showed the protein expression of knockdown of IL-1RA in HSC-3 or Ca9-22 cells using shRNA clones #1 and #2 that had a better knockdown efficiency compared to shRNA clones #3 and #4 in (B), and the protein expression of overexpression of IL-1RA in OECM-1 cells carrying a vector that expresses full-length human IL1RN (Accession: NM_173841.2). GAPDH, glyceraldehyde-3-phosphate dehydrogenase; shLuc, knockdown of firefly luciferase; shIL-1RA, knockdown of IL-1RA; EV, empty vector; IL-1RA-OE, overexpression of IL-1RA.

## Slide 2
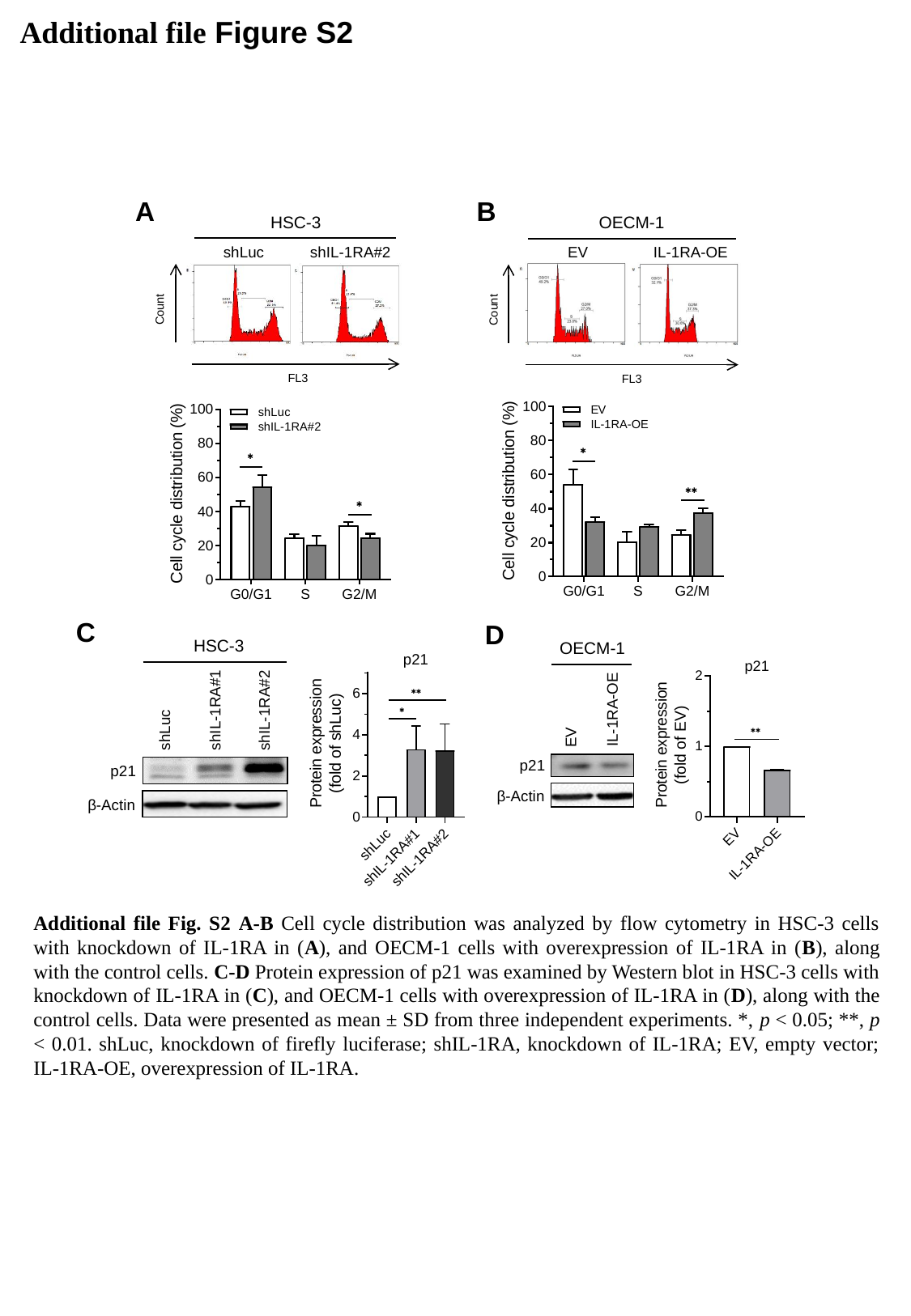

Additional file Figure S2
A
B
HSC-3
OECM-1
shLuc
shIL-1RA#2
EV
IL-1RA-OE
Count
Count
FL3
FL3
C
D
HSC-3
OECM-1
IL-1RA-OE
shIL-1RA#1
shIL-1RA#2
EV
shLuc
p21
p21
β-Actin
β-Actin
Additional file Fig. S2 A-B Cell cycle distribution was analyzed by flow cytometry in HSC-3 cells with knockdown of IL-1RA in (A), and OECM-1 cells with overexpression of IL-1RA in (B), along with the control cells. C-D Protein expression of p21 was examined by Western blot in HSC-3 cells with knockdown of IL-1RA in (C), and OECM-1 cells with overexpression of IL-1RA in (D), along with the control cells. Data were presented as mean ± SD from three independent experiments. *, p < 0.05; **, p < 0.01. shLuc, knockdown of firefly luciferase; shIL-1RA, knockdown of IL-1RA; EV, empty vector; IL-1RA-OE, overexpression of IL-1RA.

## Slide 3
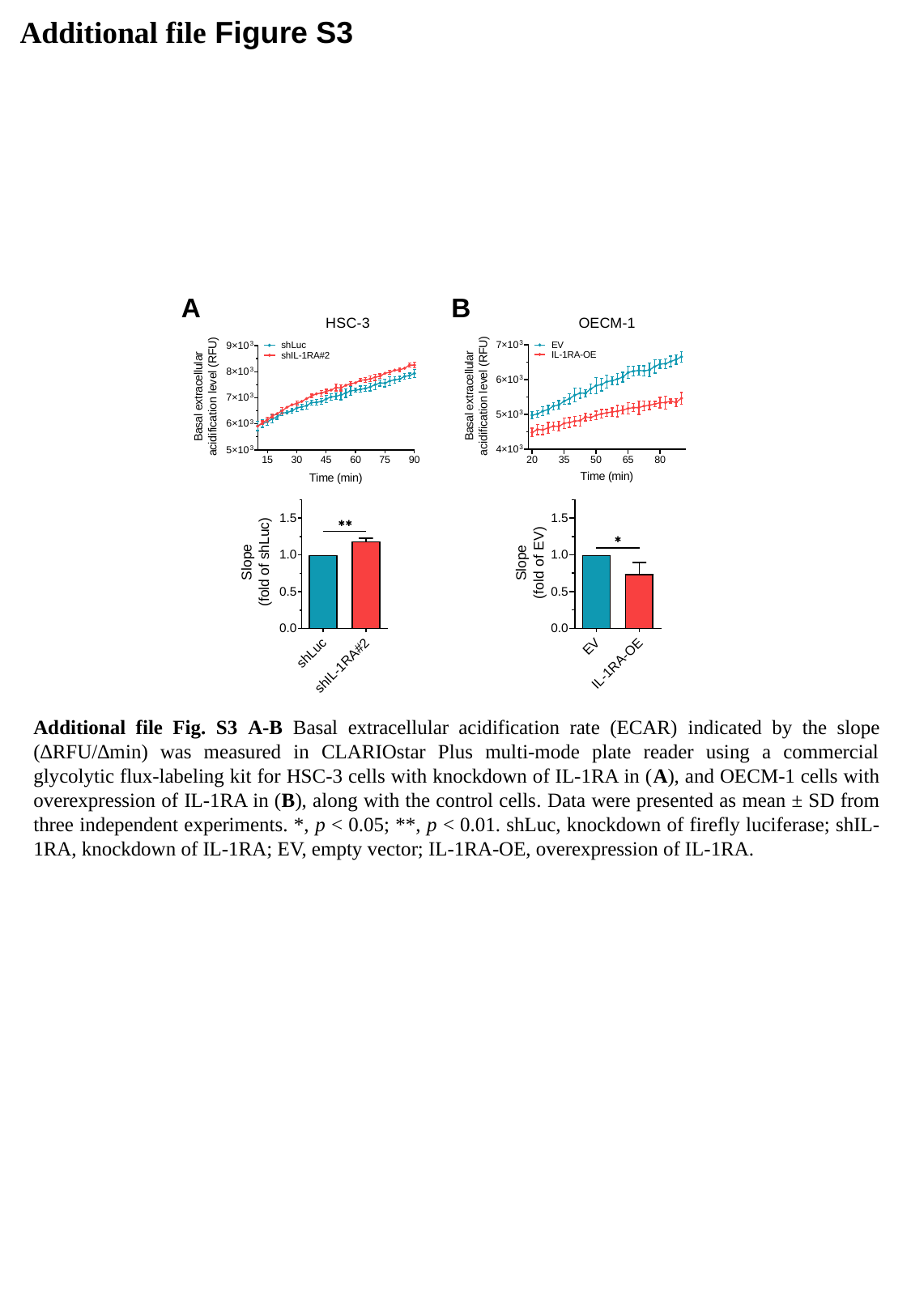

Additional file Figure S3
B
A
Additional file Fig. S3 A-B Basal extracellular acidification rate (ECAR) indicated by the slope (∆RFU/∆min) was measured in CLARIOstar Plus multi-mode plate reader using a commercial glycolytic flux-labeling kit for HSC-3 cells with knockdown of IL-1RA in (A), and OECM-1 cells with overexpression of IL-1RA in (B), along with the control cells. Data were presented as mean ± SD from three independent experiments. *, p < 0.05; **, p < 0.01. shLuc, knockdown of firefly luciferase; shIL-1RA, knockdown of IL-1RA; EV, empty vector; IL-1RA-OE, overexpression of IL-1RA.

## Slide 4
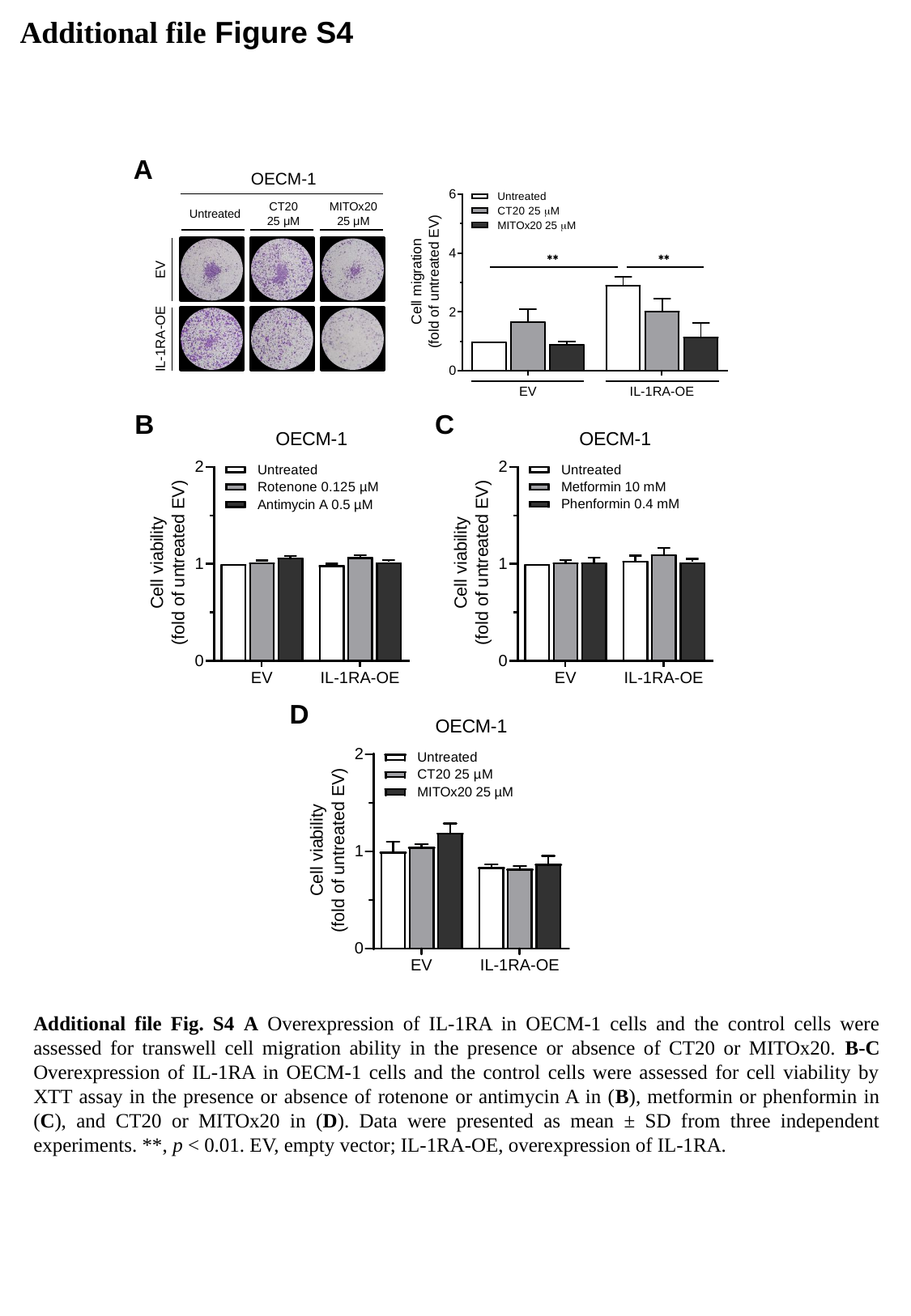

Additional file Figure S4
A
OECM-1
CT20
25 μM
MITOx20
25 μM
Untreated
EV
IL-1RA-OE
B
C
D
Additional file Fig. S4 A Overexpression of IL-1RA in OECM-1 cells and the control cells were assessed for transwell cell migration ability in the presence or absence of CT20 or MITOx20. B-C Overexpression of IL-1RA in OECM-1 cells and the control cells were assessed for cell viability by XTT assay in the presence or absence of rotenone or antimycin A in (B), metformin or phenformin in (C), and CT20 or MITOx20 in (D). Data were presented as mean ± SD from three independent experiments. **, p < 0.01. EV, empty vector; IL-1RA-OE, overexpression of IL-1RA.

## Slide 5
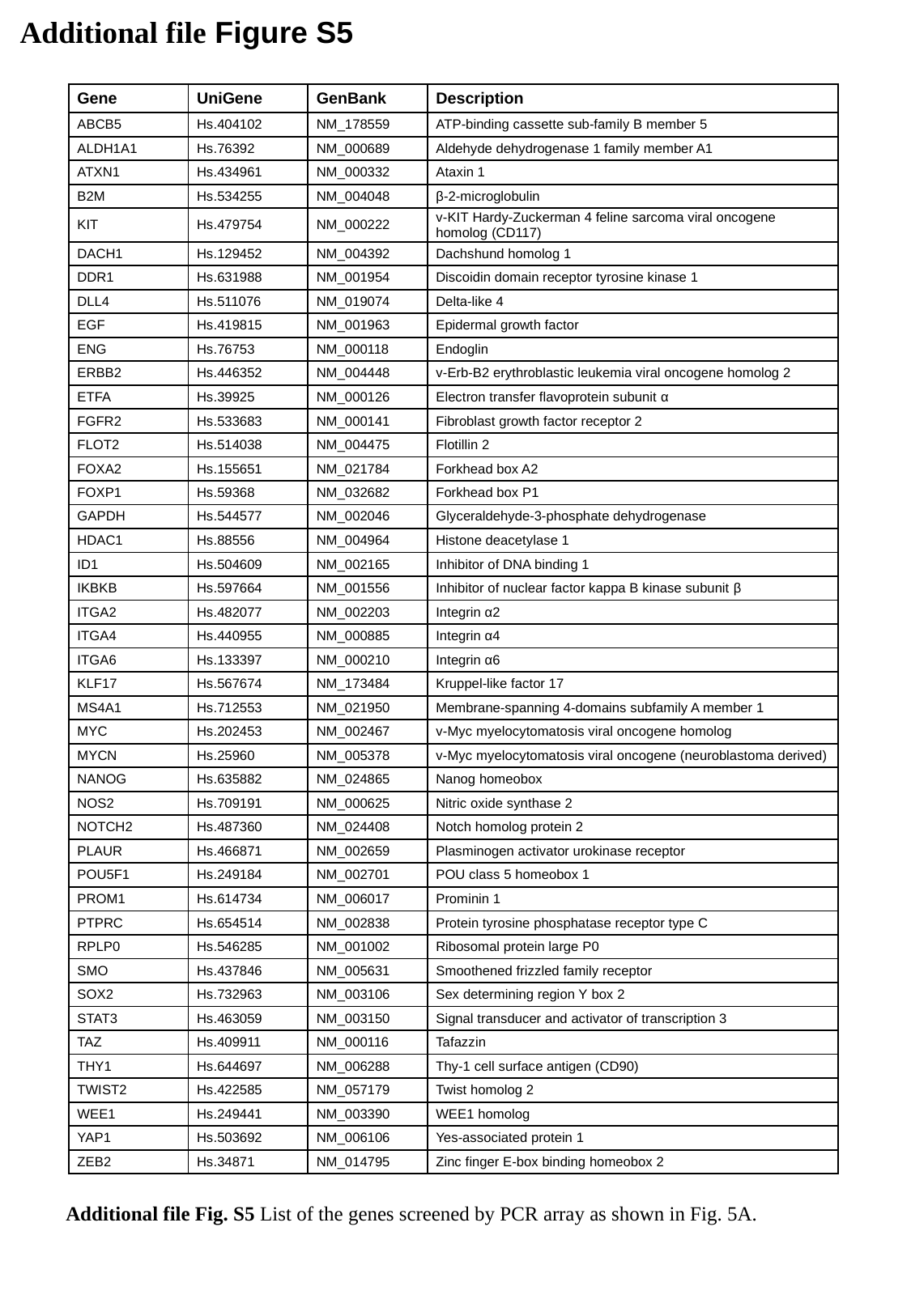

Additional file Figure S5
| Gene | UniGene | GenBank | Description |
| --- | --- | --- | --- |
| ABCB5 | Hs.404102 | NM\_178559 | ATP-binding cassette sub-family B member 5 |
| ALDH1A1 | Hs.76392 | NM\_000689 | Aldehyde dehydrogenase 1 family member A1 |
| ATXN1 | Hs.434961 | NM\_000332 | Ataxin 1 |
| B2M | Hs.534255 | NM\_004048 | β-2-microglobulin |
| KIT | Hs.479754 | NM\_000222 | v-KIT Hardy-Zuckerman 4 feline sarcoma viral oncogene homolog (CD117) |
| DACH1 | Hs.129452 | NM\_004392 | Dachshund homolog 1 |
| DDR1 | Hs.631988 | NM\_001954 | Discoidin domain receptor tyrosine kinase 1 |
| DLL4 | Hs.511076 | NM\_019074 | Delta-like 4 |
| EGF | Hs.419815 | NM\_001963 | Epidermal growth factor |
| ENG | Hs.76753 | NM\_000118 | Endoglin |
| ERBB2 | Hs.446352 | NM\_004448 | v-Erb-B2 erythroblastic leukemia viral oncogene homolog 2 |
| ETFA | Hs.39925 | NM\_000126 | Electron transfer flavoprotein subunit α |
| FGFR2 | Hs.533683 | NM\_000141 | Fibroblast growth factor receptor 2 |
| FLOT2 | Hs.514038 | NM\_004475 | Flotillin 2 |
| FOXA2 | Hs.155651 | NM\_021784 | Forkhead box A2 |
| FOXP1 | Hs.59368 | NM\_032682 | Forkhead box P1 |
| GAPDH | Hs.544577 | NM\_002046 | Glyceraldehyde-3-phosphate dehydrogenase |
| HDAC1 | Hs.88556 | NM\_004964 | Histone deacetylase 1 |
| ID1 | Hs.504609 | NM\_002165 | Inhibitor of DNA binding 1 |
| IKBKB | Hs.597664 | NM\_001556 | Inhibitor of nuclear factor kappa B kinase subunit β |
| ITGA2 | Hs.482077 | NM\_002203 | Integrin α2 |
| ITGA4 | Hs.440955 | NM\_000885 | Integrin α4 |
| ITGA6 | Hs.133397 | NM\_000210 | Integrin α6 |
| KLF17 | Hs.567674 | NM\_173484 | Kruppel-like factor 17 |
| MS4A1 | Hs.712553 | NM\_021950 | Membrane-spanning 4-domains subfamily A member 1 |
| MYC | Hs.202453 | NM\_002467 | v-Myc myelocytomatosis viral oncogene homolog |
| MYCN | Hs.25960 | NM\_005378 | v-Myc myelocytomatosis viral oncogene (neuroblastoma derived) |
| NANOG | Hs.635882 | NM\_024865 | Nanog homeobox |
| NOS2 | Hs.709191 | NM\_000625 | Nitric oxide synthase 2 |
| NOTCH2 | Hs.487360 | NM\_024408 | Notch homolog protein 2 |
| PLAUR | Hs.466871 | NM\_002659 | Plasminogen activator urokinase receptor |
| POU5F1 | Hs.249184 | NM\_002701 | POU class 5 homeobox 1 |
| PROM1 | Hs.614734 | NM\_006017 | Prominin 1 |
| PTPRC | Hs.654514 | NM\_002838 | Protein tyrosine phosphatase receptor type C |
| RPLP0 | Hs.546285 | NM\_001002 | Ribosomal protein large P0 |
| SMO | Hs.437846 | NM\_005631 | Smoothened frizzled family receptor |
| SOX2 | Hs.732963 | NM\_003106 | Sex determining region Y box 2 |
| STAT3 | Hs.463059 | NM\_003150 | Signal transducer and activator of transcription 3 |
| TAZ | Hs.409911 | NM\_000116 | Tafazzin |
| THY1 | Hs.644697 | NM\_006288 | Thy-1 cell surface antigen (CD90) |
| TWIST2 | Hs.422585 | NM\_057179 | Twist homolog 2 |
| WEE1 | Hs.249441 | NM\_003390 | WEE1 homolog |
| YAP1 | Hs.503692 | NM\_006106 | Yes-associated protein 1 |
| ZEB2 | Hs.34871 | NM\_014795 | Zinc finger E-box binding homeobox 2 |
Additional file Fig. S5 List of the genes screened by PCR array as shown in Fig. 5A.

## Slide 6
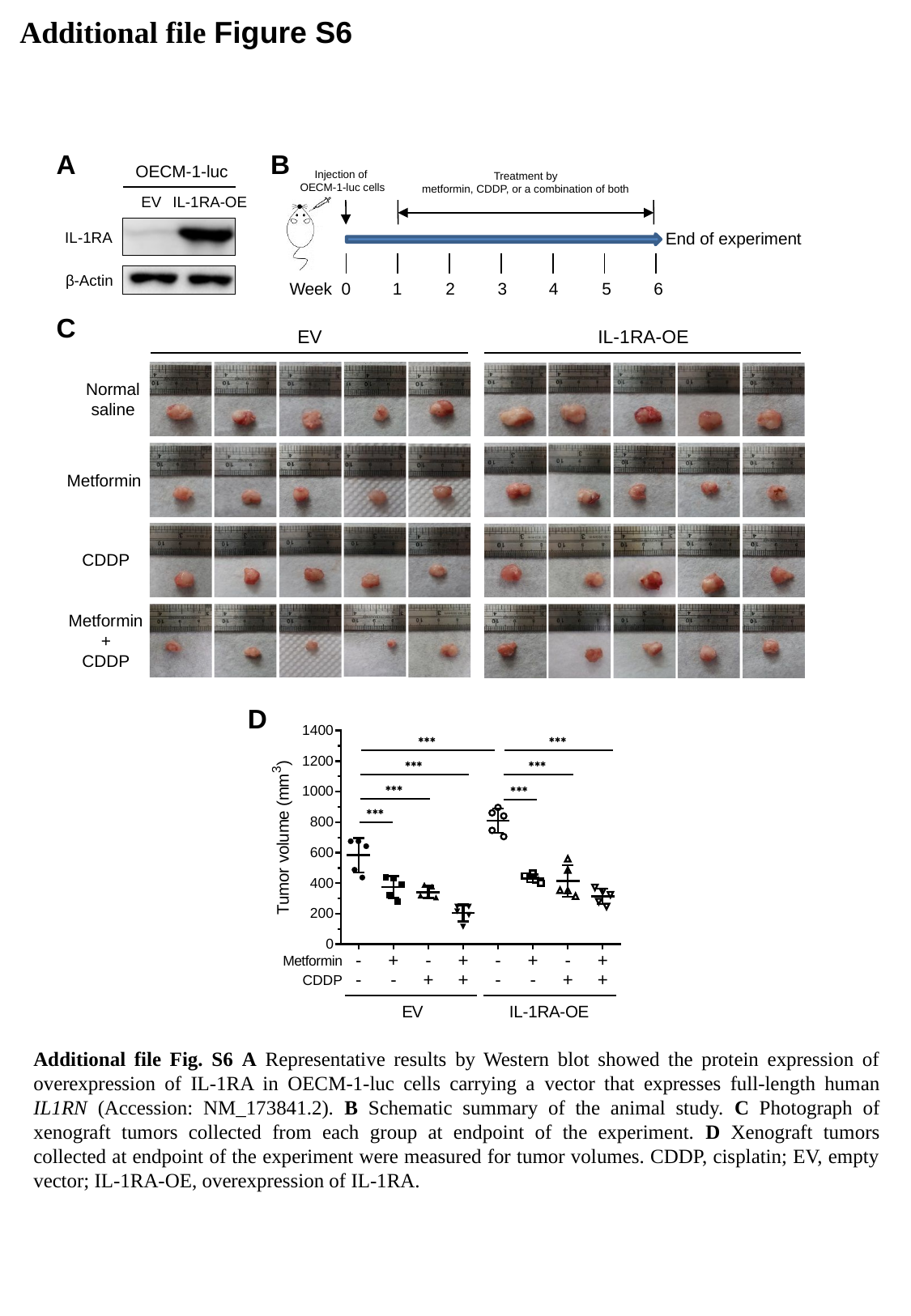

Additional file Figure S6
A
B
OECM-1-luc
Injection of
OECM-1-luc cells
Treatment by
metformin, CDDP, or a combination of both
IL-1RA-OE
EV
End of experiment
IL-1RA
| | | | | | |
| --- | --- | --- | --- | --- | --- |
β-Actin
Week 0 1 2 3 4 5 6
C
EV
IL-1RA-OE
Normal
saline
Metformin
CDDP
Metformin
+
CDDP
D
Additional file Fig. S6 A Representative results by Western blot showed the protein expression of overexpression of IL-1RA in OECM-1-luc cells carrying a vector that expresses full-length human IL1RN (Accession: NM_173841.2). B Schematic summary of the animal study. C Photograph of xenograft tumors collected from each group at endpoint of the experiment. D Xenograft tumors collected at endpoint of the experiment were measured for tumor volumes. CDDP, cisplatin; EV, empty vector; IL-1RA-OE, overexpression of IL-1RA.
